# Effect of Orthodontic Brackets on the Accuracy of Apex Locators: A Pilot Study

**DOI:** 10.1155/2021/6615560

**Published:** 2021-04-24

**Authors:** Özgür Genç Şen, Özgür İlke Ulusoy, Yelda Nayır Paltun, Çağrı Ulusoy

**Affiliations:** ^1^Department of Endodontics, Faculty of Dentistry, Yüzüncü Yıl University, Van, Turkey; ^2^Department of Endodontics, Faculty of Dentistry, Gazi University, Ankara, Turkey; ^3^Ministry of Health, Ankara, Turkey; ^4^Department of Orthodontics, Faculty of Dentistry, Gazi University, Ankara, Turkey

## Abstract

The purpose of this study was to evaluate the effect of metal orthodontic brackets on the accuracy of electronic apex locator (EAL). The actual canal lengths (ACL) of 40 mandibular incisor teeth were determined. Then, the teeth were randomly divided into two groups (*n* = 20). Orthodontic metal brackets were applied in the first group, and no brackets, in the second group. The working length of each tooth was measured with an EAL under 3 test conditions according to the distance between the lip clip and sample tooth. Data were analyzed using one-way repeated measures analysis of variance and Tukey's post hoc tests (*p* = 0.05). In the bracketed samples, when the lip clip was located at 1 cm and 2 cm from the samples. The mean differences between the EAL measurements and ACLs were statistically higher than those when the samples were located 3 cm from the lip clip (*p* < 0.05). There were also statistically significant differences between EAL measurements and ACLs in the bracketed samples located 1 and 2 cm from the lip clip (*p* < 0.05). In the nonbracketed group, the differences between EAL measurements and ACLs were not statistically significant in the samples located 1, 2, and 3 cm from the lip clip (*p* > 0.05). Use of orthodontic metal brackets can negatively influence the accuracy of the electronic apex locator when the distance between the lip clip and bracket was short. A minimum of 3 cm distance should be kept between the lip clip and tooth in order to make consistent electronic measurements.

## 1. Introduction

The prevalence of orthodontic treatment has increased among adults in recent years. The use of fixed orthodontic appliances such as brackets and wires can result in periodontal or endodontic diseases by increasing bacterial colonization [1]. Moreover, orthodontic movement is suggested as a predisposing factor for the initiation of endodontic problems such as root resorption [2, 3]. In these clinical situations, when the dental pulp becomes necrotic, the root canal contents should be eliminated as soon as possible to prevent bacterial stimulation of the resorption lesions. However, the orthodontic apparatuses applied on teeth may sometimes complicate root canal treatment procedures.

Accurate determination of working length prior to the instrumentation and obturation of root canals is an important step for a successful outcome of root canal treatment. For this purpose, methods including tactile sensation, radiographic examination, and the use of electronic apex locators (EALs) are used for the establishment of apical constriction, which is generally accepted as the terminal point of root canal treatment [4, 5]. Periapical radiographs to assess the apical level for root canal treatment provide valuable information and have been used for many years [5, 6]. However, EALs have been widely used in daily clinical practice not only to eliminate the radiation exposure from radiography but also to benefit from their practical application.

Briefly, electronic apex locators work based on the electrical conductivity between the periodontal membrane and the oral mucosa. When the current passes through the periodontal ligament to the oral mucous membrane via a lip clip and attached file, the electric circuit is closed [7]. However, the presence of electrolytes negatively influences first- and second-generation EALs to accurately detect the apical foramen [6, 8]. Root ZX, a third-generation apex locator, has the ability to measure the quotient between the impedance of 2 frequencies (0.4 and 8 kHz) and is considered as the gold standard EAL [6, 8]. Despite their popularity, the use of apex locators has some limitations. The effectiveness of apex locators for working length determination has been suggested to be influenced by root canal irrigants, metallic restorations, saliva, and interappointment medications such as calcium hydroxide [6, 9–12].

To the authors' knowledge, there is no study evaluating the impact of orthodontic metal bracket use on the effectiveness of working length determination by EALs. Therefore, the aim of this pilot study is 2-fold: (1) to investigate the accuracy of Root ZX on the establishment of the working length of teeth with metal orthodontic brackets and (2) to assess the effect of the distance between the bracket and lip clip on the consistency of EAL measurements. The null hypothesis was that the presence of orthodontic metal brackets would not influence the electronic working length measurements.

## 2. Material and Methods

The ethical clearance for this study was obtained from the Institutional Ethics Committee of Van Yüzüncü Yıl University (B.30.2.YYU.0.0.00.00/29). Freshly extracted mandibular incisor teeth with similar dimensions were selected. Radiographs were taken in buccolingual and mesiodistal directions, and the teeth were examined under 16x magnification (Leica M320, Leica Microsystems, Wetzlar, Germany) to confirm that they had mature apices, single canals, and no cracks-fractures or resorptions. The sample size calculation was based on a previous research [13], and a clinically significant 0.28 difference of EAL measurement with a standard deviation of 0.12, a significance level of 0.05, and a power of 90%. This gave a minimum sample size of 30 teeth [13]. Forty teeth meeting the inclusion criteria were cleaned of calculus and soft tissue remnants and then stored in 0.1% thymol solution at +4°C until the experiment was started. Conventional endodontic access cavities were prepared. The incisal surfaces of the teeth were flattened with a high-speed bur to create a reliable reference point for the measurements. A size #10 K-file was inserted into the root canals of the teeth until its tip was visible under 10x magnification. The distance between the file tip and the silicon stop, which was fixed to a reliable reference point, was measured using a digital caliper (Mitutoyo, Miyazaki, Japan). We subtracted 0.5 mm from this length and recorded it as “actual canal length (ACL).” After all ACLs were determined, the teeth were randomly divided into 2 main groups:

*Bracketed group (n* = 20): metal brackets (Generus series, GAC, Bohemia, NY, USA) were applied onto the surfaces of the teeth. Prior to bonding of the brackets, 37% phosphoric acid gel (Scotchbond Universal Etchant, 3M ESPE, St. Paul, MN, USA) was applied to the enamel surface of all teeth for 30 seconds. The teeth were then rinsed with water for 20 seconds and dried with an air spray until the etched surfaces appeared chalky white. The metal brackets were bonded using an orthodontic adhesive resin (Transbond XT, 3M, Monrovia, CA, USA). The brackets were positioned on the center of the buccal tooth surface with sufficient pressure. The excess adhesive was then removed from the margins of the base of the bracket using a scaler before polymerization. Adhesive was light-cured with an LED light-curing unit (3M ESPE, St. Paul, MN, USA) for 20 seconds.

*Nonbracketed group (n* = 20): no brackets were used.

**Figure 1:**
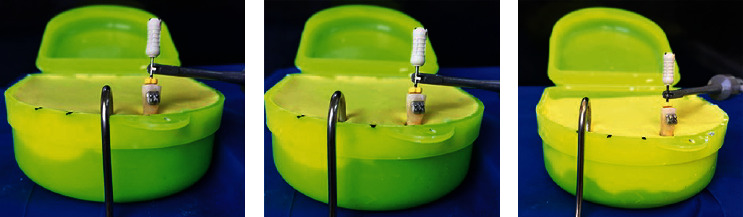
Electronic working length measurements under 3 test conditions: (a) 1 cm distance to the labial clip, (b) 2 cm, and (c) 3 cm.

Each tooth was embedded separately into freshly mixed alginate (Hydrogum, Zhermack, SpA, Rovigo, Italy) in a plastic orthodontic retainer box immediately before electronic measurement. Root canals were irrigated with 2 mL 2.5% sodium hypochlorite (NaOCl), and the pulp chambers were gently dried with cotton pellets. The lip clip electrode was attached to the apex locator, and the other electrode was attached to a #15 K-file (Dentsply Maillefer, Tulsa, OK, USA). The working length measurements of the root canals of each tooth were obtained using an EAL (Root ZX, J. Morita, Irvine, CA, USA) under the following 3 test conditions:

1. The lip clip of the EAL was embedded into alginate, keeping the distance of 1 cm from the sample tooth (Figure 1(a))
2. The lip clip of the EAL was embedded into alginate, keeping the distance of 2 cm from the sample tooth (Figure 1(b))
3. The lip clip of the EAL was embedded into alginate, keeping the distance of 3 cm from the sample tooth (Figure 1(c))

All the test apparatus was kept moist using distilled water in order to simulate the wet environment of the oral cavity throughout the experiment. The file was inserted into the canal until the apex locator indicated “apex.” After adjusting the silicon stopper of the file to a predetermined reference point, the file was removed from the canal and the distance from the file tip to the silicon stopper was measured using the same digital caliper. Thereafter, 0.5 mm was subtracted from these measurements, and the values were recorded. Each measurement was repeated three times, and the average value was recorded.

A single researcher performed all the measurements. Before performing the measurement in a new test condition, previous measurement values were put in a closed envelope and kept blinded for the remainder of the study.

### 2.1. Statistical Analysis

Statistical analysis was performed using SPSS 13.0 for Windows (SPSS Inc., Chicago, IL, USA). The normality of the data was confirmed by the Kolmogorov-Smirnov test. The data of the bracketed and nonbracketed groups were separately analyzed using repeated measures one-way analysis of variance for significant differences. Tukey's post hoc test was used for pairwise comparisons. Statistical significance was set at *p* = 0.05.

## 3. Results

Table 1 shows the mean differences between the electronic apex locator measurements and actual canal lengths (ACLs) under different test conditions.

In the metal-bracketed samples, the mean differences between the EAL measurements and ACLs were 0.387 ± 0.11 and 0.292 ± 0.071, respectively, when the lip clip was located at 1 cm and 2 cm from the samples. These mean differences were statistically higher than those when the samples were located 3 cm from the lip clip (0.08 ± 0.059) (*p* < 0.05). There were also statistically significant differences between EAL measurements and ACLs in the bracketed samples located 1 and 2 cm from the lip clip (*p* < 0.05). On the other hand, there was no statistically significant difference between the EAL measurements and ACLs in the samples that were placed 3 cm from the lip clip (*p* > 0.05).

In the nonbracketed group, the differences between EAL measurements and ACLs were not statistically significant in the samples located 1, 2, and 3 cm from the lip clip (*p* > 0.05). There was also no statistically significant difference in the three test conditions (1, 2, and 3 cm) regarding the mean differences between EAL measurements and ACLs (*p* > 0.05).

## 4. Discussion

Electronic apex locators are widely used by dental practitioners to detect precise working lengths during root canal treatment. However, their effectiveness can be influenced by factors such as root canal contents, metallic restorations, and saliva [6, 9, 10]. Metallic restorations can behave as conductors and cause short circuiting, which can affect EAL measurements [6]. Similarly, the use of orthodontic brackets in a highly liquid environment can lead to an inconsistent measurement of apex locators in working length determination. Due to the lack of knowledge regarding the effect of orthodontic brackets on the accuracy of EALs, we cannot directly compare our results with those of other studies.

In the present study, the use of metallic brackets negatively influenced the electronic working length determination. Correspondingly, in a study by El Ayouti et al. [5], more consistent results were obtained from teeth with nonmetallic restorations than from teeth with metallic restorations, although the difference was not statistically significant. They also reported no significant difference between RayPex 5 and Root ZX regarding working length measurement accuracy. In the present study, our primary aim was to assess the effect of orthodontic bracket use on the accuracy of electronic working length determination; therefore, only Root ZX was used, as it has previously been considered the “gold standard” by earlier research [6]. However, the results would be different if another type of apex locator was used, which may be considered a limitation of the current study.

Orthodontic tooth movement is accepted as one of the predisposing factors of root resorptions [14]. Internal or external root resorptions can be initiated during orthodontic therapy due to damage to the precementum or predentin [14, 15]. When the resorption process involves inflamed pulp, the necrotic root canal contents, which may stimulate the resorption process, should be removed as soon as possible. According to the results of the present study, to achieve a successful working length determination of teeth with metal brackets, the lip clip should be placed at least 3 cm from the bracket-applied teeth. However, it is difficult to maintain this distance in real clinical conditions. Therefore, different working length determination methods other than electronic apex locators (e.g., radiographical examination) are preferred in the root canal treatment of teeth with metal brackets. Alternatively, metal brackets should be removed before starting the root canal treatment. In addition, on the basis of the present results, nonmetallic bracket types instead of metallic bracket types should be used in the orthodontic treatment of patients with poor oral hygiene, who may require further root canal treatment. These issues are important contributions to the dental literature.

*In vitro* studies researching the effectiveness of EALs use different electroconductive materials, such as alginate, gelatin, or agar-agar solutions [9, 10, 16, 17]. Alginate has been shown to be a more reliable embedding medium than other materials [18, 19]. In the present study, alginate was used as the medium to mimic the periodontal ligament due to its colloidal consistency and appropriate electroconductivity [18]. To prevent desiccation, alginate was freshly mixed immediately before electronic measurement of each sample.

In the current study, to assess the influence of brackets on the consistency of working length determination, the lip clip of the EAL was placed at different distances from the sample tooth with brackets. More accurate working length measurements were derived from the bracket-applied samples that were placed at a distance of 3 cm from the lip clip than from those placed at a distance of 1 cm. In other words, close proximity of metal brackets to the lip clip of the electronic apex locator may lead to inconsistent working length determination. In the experimental setting of the present study, there was no contact between the lip file of the apex locator and the metallic bracket which can alter the working length measurement through a short-circuiting mechanism. However, the presence of a highly liquid environment simulating intraoral areas full of saliva could have increased the risk of short circuiting and interference even if there was no contact.

In the present *in vitro* experimental set, the working length measurement was performed on only one bracketed tooth; therefore, the role of the arch wire as an electrical conductor could not be evaluated. However, in real clinical conditions, more than one tooth contains brackets and wires that are used to connect them. This can be considered a limitation of this study. Another issue that should be addressed is that the lack of rubber-dam isolation could have resulted in the alteration of the working length measurements in the current research because rubber dams could prevent short circuiting and reduce the measurement deviation. However, achieving successful adaptation of rubber-dam clamps to bracketed teeth may be difficult in clinical practice. Moreover, metallic clamps of rubber dams may also influence the accuracy of EAL readings, similar to metallic brackets. The limited sample size of the present study, which can also influence the results, can be considered another limitation. Further research carried out in real clinical situations and undertaken with a larger sample size are needed to assess the consistency of apex locators in the working length estimation of teeth with orthodontic brackets, which can improve the accuracy of the present results.

## 5. Conclusion

Within the limitations of this study, it can be concluded that use of metal brackets can influence the accurate reading of EALs during working length determination of root canals in teeth having orthodontic therapy.

## Figures and Tables

**Table 1:** The mean differences between the values obtained with the electronic apex locator under different test conditions and the actual lengths (mm).

Labial clip-tooth distance	Bracketed teeth (*n* = 20)		Non-bracketed teeth (*n* = 20)	
	Mean ± SD	*p* ^∗^	Mean ± SD	*p*
1 cm	0.387 ± 0.11	*0.014*	−0.034 ± 0.042	0.43
2 cm	0.292 ± 0.071	*0.004*	−0.048 ± 0.038	0.223
3 cm	0.08 ± 0.059	1.00	−0.021 ± 0.037	0.581

Italic characters show the statistically significant *p* values (*p* < 0.05). Minus sign indicates measurements short of the actual length.

## Data Availability

The research article data used to support the findings of this study are included within the article.
